# Does Transcranial Direct Current Stimulation Combined with Peripheral Electrical Stimulation Have an Additive Effect in the Control of Hip Joint Osteonecrosis Pain Associated with Sickle Cell Disease? A Protocol for a One-Session Double Blind, Block-Randomized Clinical Trial

**DOI:** 10.3389/fnhum.2017.00633

**Published:** 2017-12-20

**Authors:** Tiago da Silva Lopes, Wellington dos Santos Silva, Sânzia B. Ribeiro, Camila A. Figueiredo, Fernanda Q. Campbell, Gildasio de Cerqueira Daltro, Antônio Valenzuela, Pedro Montoya, Rita de C. S. Lucena, Abrahão F. Baptista

**Affiliations:** ^1^Health and Functionality Study Group, Federal University of Bahia, Salvador, Brazil; ^2^Graduate Program in Medicine and Health, Federal University of Bahia, Salvador, Brazil; ^3^Health Section, Adventist Faculty of Bahia, Cachoeira, Brazil; ^4^Department of Bioregulation, Federal University of Bahia, Salvador, Brazil; ^5^Complexo Hospitalar Universitário Professor Edgard Santos, Salvador, Brazil; ^6^Physical Therapy, Loma Linda University, Loma Linda, CA, United States; ^7^Research Institute of Health Sciences (IUNICS), University of the Balearic Islands, Palma, Spain; ^8^Center for Mathematics, Computation and Cognition, Federal University of ABC, São Bernardo do Campo, Brazil

**Keywords:** neuromodulation, electroencephalography, sickle cell disease, BDNF, TNF, tDCS, peripheral electrical stimulation

## Abstract

Chronic pain in Sickle Cell Disease (SCD) is probably related to maladaptive plasticity of brain areas involved in nociceptive processing. Transcranial Direct Current Stimulation (tDCS) and Peripheral Electrical Stimulation (PES) can modulate cortical excitability and help to control chronic pain. Studies have shown that combined use of tDCS and PES has additive effects. However, to date, no study investigated additive effects of these neuromodulatory techniques on chronic pain in patients with SCD. This protocol describes a study aiming to assess whether combined use of tDCS and PES more effectively alleviate pain in patients with SCD compared to single use of each technique. The study consists of a one-session double blind, block-randomized clinical trial (NCT02813629) in which 128 participants with SCD and femoral osteonecrosis will be enrolled. Stepwise procedures will occur on two independent days. On day 1, participants will be screened for eligibility criteria. On day 2, data collection will occur in four stages: sample characterization, baseline assessment, intervention, and post-intervention assessment. These procedures will last ~5 h. Participants will be divided into two groups according to homozygous for S allele (HbSS) (*n* = 64) and heterozygous for S and C alleles (HbSC) (*n* = 64) genotypes. Participants in each group will be randomly assigned, equally, to one of the following interventions: (1) active tDCS + active PES; (2) active tDCS + sham PES; (3) sham tDCS + active PES; and (4) sham tDCS + sham PES. Active tDCS intervention will consist of 20 min 2 mA anodic stimulation over the primary motor cortex contralateral to the most painful hip. Active PES intervention will consist of 30 min sensory electrical stimulation at 100 Hz over the most painful hip. The main study outcome will be pain intensity, measured by a Visual Analogue Scale. In addition, electroencephalographic power density, cortical maps of the *gluteus maximus* muscle elicited by Transcranial Magnetic Stimulation (TMS), serum levels of Brain-derived Neurotrophic Factor (BDNF), and Tumor Necrosis Factor (TNF) will be assessed as secondary outcomes. Data will be analyzed using ANOVA of repeated measures, controlling for confounding variables.

## Introduction

Sickle-cell disease (SCD) refers to the group of hemoglobinopathies in which hemoglobin S plays a relevant role. The most prevalent genotypes are homozygous for S allele (HbSS) and heterozygous for S and C alleles (HbSC). Severity of SCD depends on different genotype, being the HbSS genotype the most severe (Rees et al., 2010). Pain is the major symptom reported by patients with SCD and it is present throughout the life span (Platt et al., 1994). The main cause of pain in SCD is the cyclic presence of ischemic vaso-occlusive events (Ballas, 2015) that can lead to severe bone tissue damage (Ballas et al., 2012), chronic joint pain syndromes (Ejindu et al., 2007) secondary to osteomyelitis, dactylitis, arthritis, and osteonecrosis (Hernigou et al., 2006; Caracas Mda et al., 2013; Flouzat-Lachaniette et al., 2016).

Chronic pain syndromes have a strong impact on quality of life of patients with SCD and lead to significant disability (Ballas, 2005). As seen in many other chronic pain syndromes, radiographic examinations relate poorly with reported pain intensity. Moreover, the effects produced by pain cannot fully explain structural injuries (Duncan et al., 2007; Bedson and Croft, 2008). Non-adaptive changes of brain areas involved in nociceptive information processing, and consequent maintenance of pain over time, may explain the disagreement between pain perception and objective clinical findings (Baliki et al., 2014). This phenomenon is termed as maladaptive plasticity (Kuner and Flor, 2016).

Although the role of maladaptive plasticity in the maintenance of chronic pain in patients with SCD is unsure, several imaging studies (Darbari et al., 2015; Campbell et al., 2016; Case et al., 2017) reveal increased functional connectivity of anterior cingulate cortex, primary and secondary somatosensory cortices (Darbari et al., 2015), as well as the periaqueductal gray matter (Case et al., 2017). These findings appear to relate with high frequency of hospital admissions (Darbari et al., 2015) and enhanced central sensitization (Campbell et al., 2016). Thus, pain in SCD seems to display similar neurobiological characteristics as other chronic pain conditions.

Some biochemical changes may explain chronic pain in SCD. Brain-derived Neurotrophic Factor (BDNF) is involved in neural regulation, maintenance, and synaptic formation, having an important role in the central nervous system plasticity (Park and Poo, 2013). BDNF levels increase in response to inflammatory conditions have been interpreted as an adaptive action related to neural protection (Schulte-Herbruggen et al., 2005; Grimsholm et al., 2008). However, higher levels of BDNF may potentiate NMDA receptors activity in the primary afferent nociceptors terminals (Chen et al., 2014), generating increased sensitization of dorsal horn neurons in response to nociceptive stimuli (Merighi et al., 2008; Biggs et al., 2010). In addition, higher levels of BNDF are associated with greater scores on central sensitization and poorer endogenous inhibitory pain control in individuals with chronic pain (Caumo et al., 2017). Serum levels of BDNF are nearly 130% higher in people with chronic joint pain (Simao et al., 2014) and positively correlated with levels of Tumor Necrosis Factor (TNF) (Grimsholm et al., 2008).

In addition to biochemical changes, electrophysiological variations are reported in brain activity of individuals with chronic pain. A systematic review (Parker et al., 2016) evaluating motor cortex excitability through Transcranial Magnetic Stimulation (TMS) identified a decrease in GABAergic intracortical inhibitory connections at the primary motor cortex (M1). This decrease is inversely correlated with serum BDNF levels (Caumo et al., 2016). Moreover, it appears to potentiate dysfunctional reorganization in M1 (Tsao et al., 2008) due to an overlap (Te et al., 2017), “blurring” (Tsao et al., 2011) and/or decreased somatotopic representation in the region (Schabrun et al., 2015). Chronic pain can be characterized also by abnormal EEG patterns, mainly a preponderance of slow brain rhythms such as delta (Walton et al., 2010), theta, and alpha (Meneses et al., 2016; Pinheiro et al., 2016). Preponderance of slow rhythms might result from significant changes in the thalamocortical loop due to sensitization of structures involved in nociceptive processing (Llinas et al., 1999).

Maladaptive brain plasticity phenomenon may underlie chronic pain and could explain why some individuals display refractory pain not responsive to pharmacological and non-pharmacological analgesic treatments (Barakat et al., 2010; New et al., 2014). Within this context, novel therapeutic strategies are necessary to reverse or diminish the effects of chronic pain. Transcranial Direct Current Stimulation (tDCS) has been investigated in several chronic pain conditions (Andrade et al., 2013; Vaseghi et al., 2014, 2015; Bolognini et al., 2015; Ngernyam et al., 2015) to modify brain maladaptive changes (Antal et al., 2010; Polania et al., 2012; Cioato et al., 2016; Sehyeon et al., 2016). This neuromodulatory technique induces neuroplasticity changes dependent on electrode polarity: anodic stimulation increases corticospinal excitability and cathodic stimulation creates an opposite effect (Nitsche et al., 2008; Nitsche and Paulus, 2011).

Lasting effects of a single tDCS session on human corticospinal excitability depend on stimulation intensity and duration (Nitsche and Paulus, 2000). For instance, healthy subjects that received 13 min of anodic tDCS stimulation over M1 displayed greater corticospinal excitability up to 90 min after the session (Nitsche and Paulus, 2000, 2001). The response to a single tDCS session on serum levels of BDNF in humans, however, remains unclear. Laboratory animal studies showed decrease of serum BDNF levels under experimental pain conditions (Spezia Adachi et al., 2015; Filho et al., 2016), which may happen in human subjects. One tDCS session can also influence EEG brain rhythms (Keeser et al., 2011; Jacobson et al., 2012) and clinical outcomes in humans, including pain (Schabrun et al., 2014). Thus, one-session tDCS protocols may be useful to generate preliminary data to give basis for prolonged use of this neuromodulatory technique.

From the clinical point of view, the effects of tDCS on chronic pain are contradictory (Luedtke et al., 2012, 2015; Wrigley et al., 2013). A recent systematic review (O'Connell et al., 2014) showed that tDCS alone provides little effect on pain control. Thus, more studies are necessary to investigate ways of enhancing its possible therapeutic benefits. Several studies have proposed an association of tDCS with other techniques, such as aerobic exercise (Mendonca et al., 2016), physical therapy (Sakrajai et al., 2014), and Peripheral Electrical Stimulation (PES) (Boggio et al., 2009; Schabrun et al., 2013, 2014; Hazime et al., 2017). These therapeutic associations assume that brain responsiveness to a particular therapy may be facilitated by techniques that alter cortical excitability (Schabrun and Chipchase, 2012). PES is a neuromodulatory technique that can induce transient changes in corticospinal excitability, depending on stimulation intensity, frequency (Chipchase et al., 2011a,b), and duration (McKay et al., 2002). PES with intensity at the sensory threshold decreases excitability, while at the motor threshold has the opposite effect (Chipchase et al., 2011a).

PES and tDCS may have an additive effect, favoring long-term potentiation or long-term depression depending on how they are combined (Muller-Dahlhaus and Ziemann, 2015). When two excitatory stimuli are associated, a null result occurs (Schabrun et al., 2013). Nonetheless, association between inhibitory and excitatory stimuli results in a synergistic effect (Boggio et al., 2009; Hazime et al., 2017). Hence, PES at the sensory threshold associated with anodic tDCS produce a sum of individual analgesic effects. As an example, association of these techniques reduced pain intensity by 36.5% while tDCS alone reduced pain by 15% in individuals with chronic pain (Boggio et al., 2009). The association of the two techniques generated better immediate effects restoring cortical representation of paraspinal muscles (Schabrun et al., 2014) and was more effective in the control of chronic low back pain than their single administration (Hazime et al., 2017).

Currently, pharmacologic interventions are the main treatment for management of chronic pain in subjects with SCD (Yawn et al., 2014). Few clinical trials evaluated the therapeutic potential of non-pharmacologic treatments (Williams and Tanabe, 2016). Consistent data suggest that the association of anodic tDCS and sensory PES is a promising therapeutic strategy for management of chronic pain. To the best of our knowledge, the study described in this protocol is the first investigating the association of these neuromodulation techniques on pain management and neurophysiological aspects of individuals with SCD. Thus, the objectives are: (1) testing the effects of anodic tDCS and PES association on pain control in subjects with SCD HbSS and HbSC genotypes; (2) and measuring the impact of the intervention on neurophysiologic parameters (electroencephalographic power density, TMS cortical mapping of the *gluteus maximus* muscle, and serum levels of BDNF and TNF) in this population.

## Objectives and hypothesis

### Primary objective

Evaluate whether a single session of anodic tDCS associated to sensory PES is superior to reduce pain intensity of individuals with SCD (HbSS and HbSC) compared to individual or sham use of these techniques.

### Secondary objective

- Evaluate the effect of a single session of anodic tDCS associated with sensory PES on the following neurophysiological variables: electroencephalographic power density, TMS cortical mapping of the *gluteus maximus* muscle, and serum levels of BDNF and TNF;
- Evaluate whether therapeutic response varies according to genotypes HbSS and HbSC.

### Hypothesis

A single session of anodic tDCS associated with sensory PES provides greater analgesic effect compared with single use of the techniques;

The association of anodic tDCS and sensory PES causes greater decrease on serum levels of BDNF and TNF and greater increase on the muscle *gluteus maximus* cortical representation compared to the individual use of the techniques;

A session combining anodic tDCS and sensory PES leads to greater decrease of electroencephalographic low frequency (delta, theta, and alpha) power density compared to the individual use of the techniques in participants with SCD.

## Methodology

### Study design, description of allocation, and blinding

**Figure 1:**
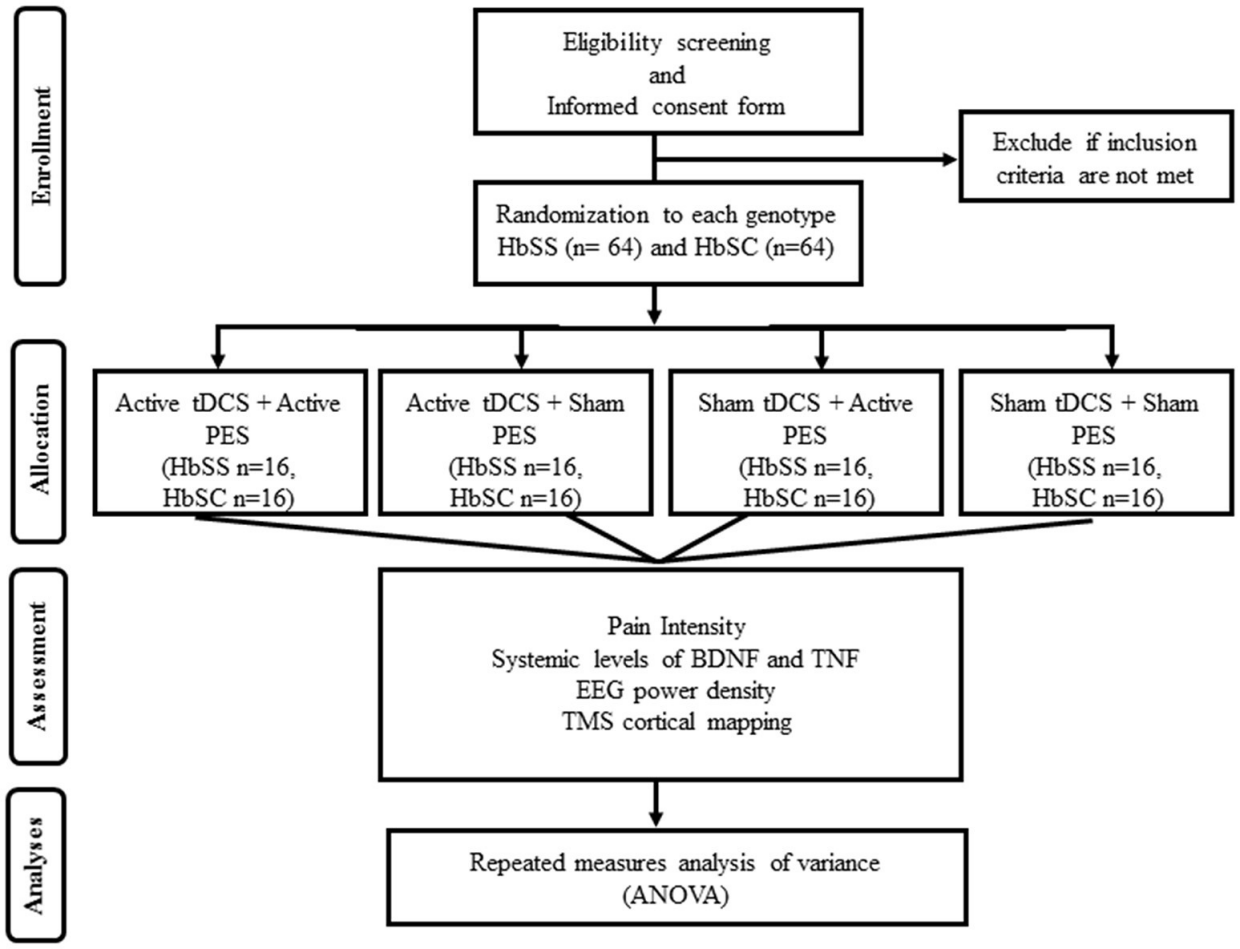
Flow chart of the study based on CONSORT criteria.

This is a clinical, parallel, controlled, block-randomized, double blind trial. The study will occur in 2 days at the Clinical Electrophysiology Laboratory of the Federal University of Bahia, Brazil. An assistant researcher not involved in any other stage of the study will generate an allocation sheet (www.randomization.com) creating four groups for each SCD genotype (HbSS and HbSC), totaling eight experimental groups. Allocation concealment will be enforced using sealed opaque envelopes listed in ascending order, and the allocation secrecy will be maintained until the end of the analyses. The allocation envelope will be opened on the day of the intervention according to the order of inclusion of the study participant. The envelope will contain one of the following interventions: (1) active tDCS + active PES (*n* = 16); (2) active tDCS + sham PES (*n* = 16); (3) sham tDCS + active PES (*n* = 16); (4) sham tDCS + sham PES (*n* = 16) (Figure 1). These interventions will be tested either in the HbSS (*n* = 64), and HbSC (*n* = 64) groups, totalizing 128 participants.

### Participants and eligibility and discontinuity criteria

Participants diagnosed with SCD will be enrolled from health units at municipalities of the 31st Regional Health Directorate (DIRES—BA) and in Reference Centers for treatment of SCD in the municipalities of Salvador and Feira de Santana, the two largest cities in the State of Bahia, Northeastern Brazil. SCD is more prevalent in this region of the country. Enrollment of participants will take place between March 2016 and December 2019. The study will be advertised in the SCD reference centers and health care facilities in the two cities. To improve adherence to the intervention protocol, all participants will receive financial aid for round-trip transportation, as well as a meal voucher. In addition, they will receive SCD care recommendations. At the end of the study, all participants will be offered the most effective intervention.

To homogenize the sample, only participants with SCD of HbSS and HbSC genotypes suffering from chronic pain secondary to femoral head osteonecrosis will participate in this study. Theses genotypes were chosen because they are more prevalent, and both have a higher prevalence of femoral head osteonecrosis in adult individuals (Milner et al., 1991). Further eligibility and discontinuity criteria of this study are listed below.

#### Inclusion criteria

- Chronic pain secondary to hip osteonecrosis of at least 6 months of duration;
- Pain intensity above 3 in a 11 points visual analogic scale (VAS);
- Individuals from both sexes, between 18 and 50 years of age.

#### Exclusion criteria

- Any contraindication to use TMS and tDCS such as: cochlear implant, cardiac pacemaker or metal implantation in the skull/brain; drug treatment that modifies the threshold of neuronal activation (i.e., antidepressant, anticonvulsant, and antipsychotic); history of seizure or epilepsy; and pregnancy;
- Neuropathic pain screened by the *Douleur Neuropathique* 4 questionnaire (DN-4) (Santos et al., 2010);
- Metal implants at the PES site;
- Occurrence of infectious disease in the week before inclusion in the study.

#### Discontinuity

- Moderate adverse effect (i.e., discomfort enough to interfere with patient's usual activities) or severe (i.e., significant impairment of the patient's usual activities or even total disability, and life threatening) during neurophysiological evaluation or intervention;
- Participant withdraws consent at any stage of the study.

### Interventions

An experienced physical therapist will manage the intervention protocols. During the intervention, participants will be comfortably seated in a chair, in silence. They will be encouraged to make no cognitive effort, such as mathematical calculations or complex reading. tDCS will be applied with a proper device (tDCS stimulator–TCT, China) connected with two silicon-sponge 5 × 7 (35 cm^2^) electrodes embedded in saline solution (0.9%). The anodic pole will be placed in the motor cortex region (M1) contralateral to the painful hip (or more painful in cases of bilateral symptoms) (locations C1 or C2, according to the 10/20 International EEG System). The cathodic pole will be placed over the contralateral supra-orbital region (locations Fp2 or Fp1, according to the 10/20 International EEG System). The 2 mA stimulation will last 20 min, ramped up and down for 30 s at the initial and final stages of stimulation. A clinical pulse generator (Endophasys, KLD Medical Products, Brazil) will be used to administer PES using 35 cm2 dischargeable electrodes located on the side of the most painful hip. The stimulation will start together with tDCS and will be held for 30 min. The intensity will be maintained at the sensory level, characterized as a comfortable intensity just under motor threshold, with pulse rate of 100 Hz and pulse duration of 200 μs.

Participants will be asked about their perception of the stimuli every 5 min during the intervention. For both tDCS and PES sham procedures, the devices will be ramped up for 30 s and then decrease until no electric current is delivered. To ensure blinding, participants will receive information that they may or may not feel the stimulation. Moreover, they will not be able to see the equipment. At the end of each intervention protocol, potential adverse effects and quality of blinding will be assessed by a self-report questionnaire.

### Clinical and sociodemographic characterization of the sample

Sample will be characterized through socio-demographic (gender, age, education level, profession, marital status, and race) and clinical data (anxiety/depression symptoms and disability index related to pain). Socio-demographic data will be collected by self-report questionnaire designed specifically for this study. Clinical data will be collected using the following instruments:

- Hospital anxiety and depression scale (HADS): HADS comprises two seven-items subscales. Subjects will rate each item using an ordinal scale varying from zero (non-existent symptom) to three (very severe symptom) (Pais-Ribeiro et al., 2007).
- Pain Disability Index (PDI): PDI is composed of seven items to assess how pain interfere in daily activities, including: family and domestic obligations, recreation, social activities, profession, sexual life, autonomy, and elementary activities indispensable to life. Subjects will rate each item using an ordinal score ranging from zero (no disability) to 10 (total disability) (Tait et al., 1990).

### Outcome measures

#### Primary outcome measure

Pain intensity will be measured by the VAS, in two positions: participant sited comfortably and then sited abducting the most painful hip. The VAS ranges from zero to 10, where zero represents no pain at all and 10 the worst imaginable pain. This evaluation will be performed pre- and post-intervention.

#### Secondary outcome measures

A. Power density of electroencephalographic frequencies

Relative power density will be measured pre- and post-intervention. An electroencephalograph (EEG Brainet 36, EMSA, Brazil) with 30 electrodes arranged according to the international system of electroencephalography 10/10 will be used in the following electrode locations: F7 T3 T5 Fp1 F3 C3 P3 O1 F8 T4 T6 Fp2 F4 C4 P4 O2 Fz Cz Pz Oz FT7 FT8 TP7 CP3 FC3 CPz FCz CP4 FC4 TP8. EEG data will be collected at a sampling rate of 600 Hz and referenced to Cz channel. Impedance will be maintained below 5 kΩ for all electrodes. The recording environment will be kept in subdued light and protected by a Faraday cage. Participants will be instructed to sit comfortably in a chair, keeping their eyes closed during EEG recording, which will occur in the two conditions described below.

a. Resting state: This condition will last 4 min, and the participant will be asked to not focus on any specific cognitive activity;
b. Kinesthetic motor imagery (Kinesthetic–MI): Kinesthetic–MI will follow the resting state recorded, divided into two distinct stages. (1) *Kinesthetic–MI of a movement in the non-painful region of the body:* After 10 s of recording, participants will hear a standardized command requesting to mentally simulate closing and opening the contralateral hand to the most painful hip side. The command to each mental simulation will be repeated eight times, being 5 s of closing movements and 10 s of opening movements of the hand, totaling 120 s. (2) *Kinesthetic–MI of a movement in the painful region of the body:* The same protocol described above will be repeated, but this time the command will be to mentally simulate the abduction and adduction of the most painful hip. Electromyographic (EMG) data of the *gluteus medius* muscle on the most painful side and flexor muscles of the fingers contralateral to the hip will be also recorded to ensure absence of real movement. After each recording, the quality of the Kinesthetic–MI will be assessed by the kinesthetic and visual imagery questionnaire (KVIQ) (Malouin et al., 2007). EEG recording will take ~1 h.

Pre-processing of EEG data will be done using the MATLAB software version 2015 and EEGlab *toolbox* version 14. The signals will be filtered offline with a bandpass between 0.5 and 45 Hz. EEG data will be segmented into epochs of 1.71 s to allow an analysis of power densities at frequencies ranging from 1.2 to 30 Hz. EEG artifacts with minimum amplitude below −750 or maximum amplitude above 750 μV will be rejected using a semi-automated protocol. EEG data with more than 33% of rejected epochs will be excluded from further analyses. After the artifact rejection protocol, all EEG data will be adjusted to have the same number of epochs.

Power density will be calculated by fast Fourier transform in each epoch and electrodes, separately for each participant. The average power densities will be grouped in delta (1.2–3.5 Hz), theta (4–7 Hz), alpha (8–12 Hz), and beta (13–30 Hz) frequency bands. Regions of interest (ROI) chosen for analysis will be computed by averaging power densities for the following groups of electrodes: frontal (F7, F3, Fp1, Fz, Fp2, F4, F8), central (FC3, C3, FCz, Cz, C4, FC4), parietal (CP3, P3, CPz, Pz, CP4, P4) temporal (FT7, T3, TP7, T5, FT8, T4, TP8, T6), and occipital (O1, Oz, O2). After obtaining absolute power densities, the relative power density will be calculated dividing electrode's values in each one of the analyzed frequencies by their values in the total power spectrum.

B. TMS cortical mapping

Cortical mapping will be assessed pre- and post-intervention. After EEG recording, each participant will be asked to lie down comfortably in a supine position on an examination table, with head and neck resting on a support. The TMS cortical mapping will be performed using a single-pulse TMS apparatus (Bi-Stim; Magstim Co. Ltd, Dyfed, UK) delivered over M1 to cover the representation of both *gluteus maximus* muscles. A figure-of-eight coil will be positioned with the handle oriented backwards and aligned to the sagittal suture, inducing a postero-anterior flow of current. One cap marked with an 8 × 7 cm grid and oriented to the vertex will be placed on the participant's head and regularly checked to guarantee placement consistency. The vertex will be marked on the intersection of interaural and nasion to inion lines according to the 10/20 international EEG system.

The stimulus intensity for mapping will be set at 120% of Active Motor Threshold (AMT) for the *gluteus maximus* muscle. AMT will be the minimum intensity at which a TMS stimulus evokes a response of 200 μV while the *gluteus maximus* muscle is contracted under a comfortable bridge position in supine. Then, the AMT will be determined using the adaptive TMS motor threshold assessment tool (MTAT 2.0). The TMS pulse will be applied every 6 s, with a total of five stimuli at each site on the 8 × 7 cm grid.

Surface electrodes Ag/AgCl (3M, USA) will be used to record EMG activity at the *gluteus maximus* muscles, bilaterally. The two registering electrodes will be placed at 50% on the line between the second sacral vertebrae (S2) and the hip greater trochanter. The positioning of the electrode will be determined after palpation of the *gluteus maximus* muscle during a moderate voluntary contraction. The reference electrode will be placed over the hip greater trochanter or S2 (Fisher et al., 2013). The EMG signals will be amplified 3,000 times, filtered, bandpassed between 1 Hz and 2 kHz, with sampling rate maintained at 4 kHz using Signal v.06 software (Cambridge Electronic Design, UK). EMG data elicited by TMS will be monitored in real time to ensure the consistency of evoked responses (Signal, Cambridge Electronic Design, UK). Mapping of the *gluteus maximus* muscle will take ~40 min.

TMS map volume and center of gravity (CoG) of both *gluteus maximus* muscles will be used as dependent variables. These parameters will be calculated, respectively, by the sum of normalized MEP amplitudes at each site and by the formula:

$$\text{CoG}=\sum {\text{V}}_{\text{iX}}{\text{X}}_{\text{i}}/\sum {\text{V}}_{\text{i}};\sum {\text{V}}_{\text{iX}}{\text{Y}}_{\text{i}}/\sum {\text{z}}_{\text{i}}$$

Where, V_i_ = mean MEP amplitude at each site with the coordinates X_i_, Y_i_

C. Serum levels of BDNF and TNF

Serum levels of BDNF and TNF will be measured pre- and post-intervention. Approximately 5 mL of blood will be collected from each study participant and stored in test tubes with anticoagulant EDTA (0.03%). This procedure will last <5 min. The blood sample will be centrifuged at 2,500 rpm for 10 min and the plasma stored at a temperature of −40°C.

At a later time, serum levels of BDNF and TNF will be quantified using enzyme-linked immunosorbent assay kits (DuoSet, R&D Systems, Minneapolis, MN). A volume of 100 μL of monoclonal antibody-based capture will be added to a 96-well plate, which will be incubated for 12 h at room temperature (RT). The wells will be washed with wash buffer (PBS/Tween) and incubated with a blocking solution (300 μL) containing PBS and bovine serum albumin (BSA) for 1 h at RT. Samples and standards will be plated and incubated for 2 h at RT. After washings, the detection monoclonal antibody will be added to the plate and incubated for 2 h at RT. Then, a streptavidin-peroxidase solution will be added and incubated for 1 h at RT.

Finally, the substrate solution (H_2_O_2_ and TMB tetramethylbenzidine) will be added to the plate and a blue color will develop within a period of 20 min. The staining reaction will be stopped by adding H_2_SO_4_ 2N, and the reading will be made on a microplate reader at 450 nm. Levels of BDNF and TNF will be expressed in pg/mL and calculated from the reference values obtained with a standard curve built with known concentrations of recombinant BDNF and TNF. Concentrations of BDNF and TNF in plasma will be quantified using commercially available antibody pairs and recombinant cytokine standards (DuoSet, R&D System, Minneapolis, MN), using sandwich enzyme-linked immunosorbent assay (ELISA) according to the manufacturer's instructions.

### Potential adverse effects

The tDCS has relatively minimal adverse effects, which include: mild tingling; itching; burning and mild pain sensation under the surface of the electrodes; fatigue; and somnolence (Poreisz et al., 2007). The main adverse effects of PES are, skin irritation and local allergic reaction (Coutaux, 2017). Nonetheless, none of these adverse events are considered to be serious. These potential adverse effects may be avoided by appropriate training in the handling of the technique. The use of TMS to evaluate the motor cortical representation of the *gluteus maximus* muscle also has some potential risks such as seizures, syncope, and headache (Rossi et al., 2009), but all are rare and will be duly clarified to participants during recruitment and signature of informed consent. The Clinical Electrophysiology Laboratory team has trained physical therapists and physicians accessible to assist in case any harm occurs to a participant. Any adverse effect that may occur during the study, even those not directly related to the study assessments and interventions, will be reported to the Institutional Review Board.

### Sample size

The sample size was calculated using the GPower software version 3.1.9.2 (Faul et al., 2007). The overall study objective is to test the hypothesis that association of anodic tDCS and sensory PES reduces pain intensity by a large percentage as opposed to use of isolated tDCS or PES in individuals with SCD. We assumed that the sample will be equally randomized into four intervention groups: (1) active tDCS + active PES; (2) active tDCS + Sham PES; (3) Sham tDCS + Active PES; and (4) Sham tDCS + Sham PES. We proceeded with the desired sample size assuming no financial or logistic limitations. The parameters used were: 80% power; 5% Type I error; effect size of 0.35 (Cohen, 1977) on the reduction of pain intensity evaluated by the VAS; four intervention groups; two genotypes (HbSS and HbSC) subgroups; and two repeated measures (pre- and post-intervention). Using these parameters yield to an estimated 64 participants for each genotype, a total *n* = 128. To increase the likelihood of achieve the computed sample size in a timely fashion, this clinical trial will be promoted through social media and posters in SCD treatment centers explaining the purpose and potential benefits of the study.

### Statistical analysis

To ensure impartiality of the results, researchers who do not participate in any stage of data collection will perform the statistical analysis. Descriptive statistic will be used to summarize demographic and clinical sample characteristics. Shapiro–Wilk test will be performed to test normality of the data. Chi-square test will be used to compare frequency distributions and one-way ANOVA or Kruskal–Wallis *H*-test will be used to compare baseline means among the four intervention groups. The main outcome of the study is pain rating measured by the VAS. For that, we will run a repeated measure analysis of variance (ANOVA) to evaluate differences among intervention groups (active tDCS + active PES; active tDCS + Sham PES; Sham tDCS + Active PES; and Sham tDCS + Sham PES), genotypes (HbSS and HbSC), and time (pre- and post-intervention). For most of the secondary outcomes (TMS volume map; TMS center of gravity; BDNF serum level; and TNF serum level) the repeated measures ANOVA will have the same three factors listed above. Relative EEG power will further include in the ANOVA the factors ROI (frontal, temporal, central, parietal, and occipital) and EEG condition (resting, Kinesthetic–MI of painful, and Kinesthetic–MI of non-painful region of the body). All analysis will be controlled for anxiety/depression symptoms. Bonferroni test will be used to correct for multiple comparisons. An α-value of 5% (*P* < 0.05) will be used to accept statistically significant differences for all analyses.

### Ethical aspects

Volunteers will receive explanation regarding their participation and the freedom to remove consent at any time during the study. They will read and receive answers to any questions before signing an Informed Consent Form (ICF), prepared according to Resolution 466/2012 of the Brazilian National Council of Health. This study has been approved by the Ethics and Research Committee (ERC) of the Adventist Institution of Bahia (CAAE No. 31237514.1.0000.0042). The study is registered at the Protocol Registration and Results System (PRS), trial number NCT02813629. Any changes in the study protocol will be informed to both the ERC and the PRS.

## Stepwise procedures

**Figure 2:**
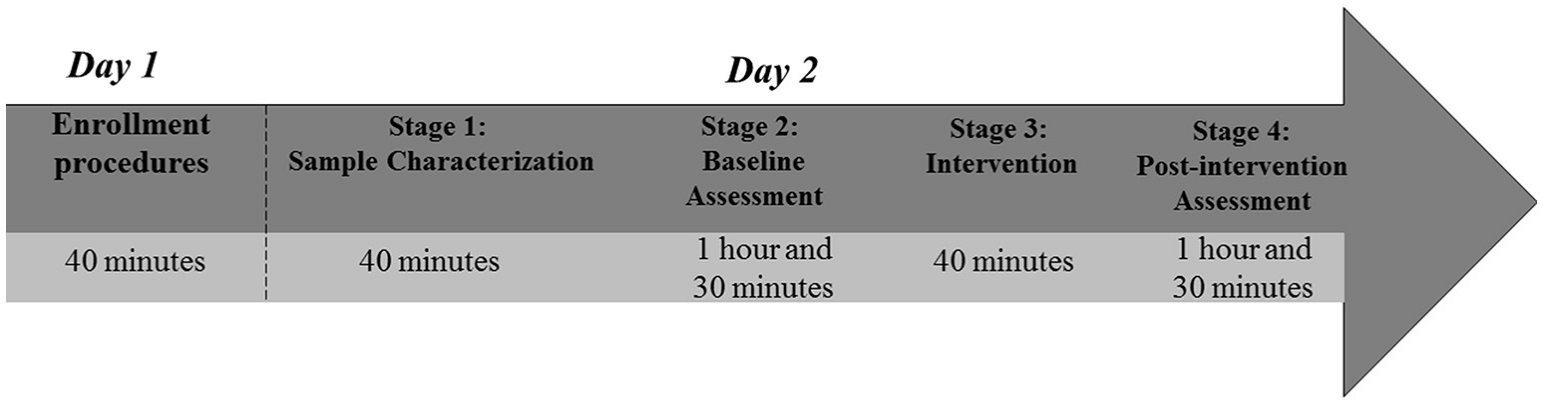
Timeline and duration of study procedures.

Study procedures will occur in 2 independent days (Figure 2).

*Day 1: Enrollment procedures*. A trained research assistant will visit SCD reference centers and health clinics to interview potential eligible subjects. Subjects interested in the study will receive detailed information about all procedures and duration of the study. Those willing to participate will be screened for eligibility criteria (40 min). Eligible subjects will receive the ICF to read. The assistant research will read aloud the ICF if requested by the subject. All doubts will be clarified and the ICF will be filled out and signed. Participants will be scheduled for data collection in the Clinical Electrophysiology Laboratory at the Federal University of Bahia.

*Day 2: Data collection*. Procedures will begin in the morning. Upon arrival of participant at the study room, an allocation envelope will be opened to determine participant's intervention group. Data collection will be divided into four stages (sample characterization, baseline assessment, intervention, and post-intervention assessment).

- *Stage 1* (Sample characterization) will last ~40 min. The researcher will explain to the participant why and how to fill the socio-demographic and clinical questionnaires. The researcher will be available to clarify any doubts and help participant, if requested;
- *Stage 2* (Baseline assessment). EEG data will be collected during resting and Kinesthetic–MI conditions. Subsequently, in another room, the participant will receive instructions for the TMS cortical mapping. Following these electrophysiological assessments, a blood sample will be collected for BDNF and TNF serum level analysis. Finally, the pain intensity will be assessed. This stage may last up to 1 h and 30 min;
- *Stage 3* (Intervention). At this stage, the participant will be seated and the researcher will position the tDCS electrodes on the scalp according to the 10/20 EEG system. Electrodes will be fixed with an elastic band, comfortably adjusted. The PES electrodes will be positioned at the most painful hip. This stage will last around 40 min;
- *Stage 4* (Post-intervention assessment). In the last part of the study, the sequence of assessments will be slightly different from Stage 2. First, pain intensity will be measured. Then, a blood sample will be collected. Blood sample must be collected earlier in this stage to reduce circadian cycle effect on BDNF and TNF serum levels (Pan et al., 1985; Begliuomini et al., 2008). Finally, EEG data and TMS cortical mapping will be collected. The final stage is expected to last up to 1 h and 30 min.

Participants may take 5–10 min-breaks between all stages. During their time in the laboratory room, participants will have snacks and light beverage available at breaks. Participants will be reimbursed for transportation costs and receive a food voucher at the end of data collection.

## Potential pitfalls and counteracting measures

This protocol was designed with the main purpose of answering the question “Does tDCS combined with PES have an additive effect in the control of osteonecrosis joint pain associated with SCD?” As in any clinical trial, risk of bias and errors exist. Several strategies will be used to reduce them:

### Potential errors

The development of this protocol involved several experts to carefully design the interventions in order to reduce systematic errors. To minimize human errors, all personnel involved in data collection will be thoroughly trained on how to use study questionnaires and instruments.

### Potential sampling biases

According to the Ficat classification (Ficat, 1985), the femoral head osteonecrosis has four radiographic stages of progressive severity. However, the study sample will not be stratified by degree of femoral osteonecrosis because the association between radiographic examinations and pain intensity is weak (Bedson and Croft, 2008). The randomization method used will likely reduce sampling biases by distributing patients at different osteonecrosis stages equally among groups (Kang et al., 2008). Including only hip joint osteonecrosis may limit the generalization to the larger population of individuals with SCD. Nonetheless, this chronic musculoskeletal pain condition has high prevalence among individuals with SCD (Milner et al., 1991; Hernigou et al., 2006).

### Potential intervention biases

Stimulation duration in this study will differ between neuromodulatory techniques, being 20 min for tDCS and 30 min for PES. The choice for a longer time of PES stimulation was based on studies demonstrating that 30 min provides significant improvement on cortical excitability (McKay et al., 2002). To avoid proficiency bias, interventions will last 30 min. Studies using neuromodulatory techniques, such as tDCS and sensory PES, have shown that one of the reliable alternatives for performing blinding is to instruct participants that they may or may not feel the stimulation (McDonnell et al., 2007; Schabrun et al., 2013, 2014). A recent study using tDCS for 30 min showed that the erythema in the supra-orbital region can occur, ranging from mild to moderate, and may interfere with the study blinding (Ezquerro et al., 2017). To avoid that, participants in this study will not be able to look at their faces during the stimulation procedure. Thus, they will not be able to recognize any erythema over their frontal region. In addition, gradual ramp up/down of stimulation intensity is considered very effective in blinding study participants (Rakel et al., 2010). Finally, equipment will be hidden in boxes to ensure that participant will be unable to see what the researcher is doing.

### Measurement biases

*Gluteus maximus* muscle TMS mapping may be difficult because of its small and deep representation in the primary motor cortex. We aim to counteract this problem using active motor threshold and contraction during TMS assessment, a method successfully used by others (Lepley et al., 2013; Te et al., 2017). Neuronavigated TMS could further help with this measurement problem, but this resource is not available at the study laboratory. Nonetheless, the original study to validate TMS cortical mapping of the *gluteus maximus* muscle used a system similar to the one available for this study (Fisher et al., 2013).

### Potential confounders

A. Pain intensity

Emotional aspects such as anxiety and depression are commonly associated with chronic pain (Keefe et al., 2004). Anxiety and depressive symptoms are associated with increased sensitivity to experimental painful stimuli in individuals with SCD (Bakshi et al., 2017). In order to control for this confounding factor, symptoms of anxiety and depression will be evaluated pre-intervention using the HADS (Pais-Ribeiro et al., 2007).

B. Serum level BDNF and TNF

Circadian rhythm has influence on BDNF and TNF serum levels. A study with healthy subjects evaluated BDNF levels every 4 h and identified a continuous decrease throughout the day (Begliuomini et al., 2008). Similarly, an animal study demonstrated that levels of TNF also varies according to the circadian rhythm (Pan et al., 1985). To reduce the influence of circadian rhythm, data collection will start at the same time in the morning. Recent studies have shown that the presence of the Val66Met BDNF gene polymorphism may influence cortical neuroplastic changes and consequently the response to tDCS (Antal et al., 2010; Di Lazzaro et al., 2015). However, the population studied in this protocol will be mostly Afro-descendant of Yoruba origin, of which only 0.9% is heterozygous for the Met allele (Aken et al., 2016).

## Criteria of authorship

To be included as an author in the articles from this study, individuals should meet the following criteria of authorship (Petroianu, 2010):

- Contribute substantially to the study conception and design; or to the acquisition, analysis, or interpretation of study data;
- Participate in the writing of this manuscript or critically examine its intellectual content.

## Author contributions

TL, AB, WS, and SR were involved in all aspects of study design and conception. TL drafted the introduction, while AB, PM, and FQC critically reviewed it. All authors contributed with the design of study methodology. WS and CF contributed with data collection and design of methodology and analysis of serum BDNF and TNF levels. FQC contributed with the calculation of sample size and designed the statistical analysis. FQC, PM, and AV reviewed the English. RL and SR contributed with conception and design of tDCS data collection and safety aspects. PM and GD contributed with selection of clinical questionnaires in the study design. PM and FQC contributed with the conception and design of the EEG data collection, pre-processing, and analysis. All authors contributed with the drafting of the work. TL, AB, PM, FQC, and CF revised the manuscript critically for important intellectual content. All authors gave final approval of the version to be published. All authors agreed to be accountable for all aspects of the work in ensuring that questions related to the accuracy or integrity of any part of the work are appropriately investigated and resolved.

### Conflict of interest statement

The authors declare that the research was conducted in the absence of any commercial or financial relationships that could be construed as a potential conflict of interest.
